# Nutritive quality of romanian hemp varieties (*Cannabis sativa L.*) with special focus on oil and metal contents of seeds

**DOI:** 10.1186/1752-153X-6-122

**Published:** 2012-10-23

**Authors:** Marcela Mihoc, Georgeta Pop, Ersilia Alexa, Isidora Radulov

**Affiliations:** 1 Faculty of Agriculture, Banat’s University of Agricultural Sciences and Veterinary Medicine, Calea Aradului 119, RO 300645, Timisoara, Romania

**Keywords:** Hempseed, Oil content, Metal content

## Abstract

**Background:**

The study aims to determine the nutritional value of hemp seed expressed by the oil content and by the concentration of metals (Ca, Mg, K, Fe, Mn, Zn and Cd), for five varieties of monoecious and dioecious hemp seeds approved in Romania, comparative with the concentration of these metals in the soil.

**Results:**

The content of oil in hempseed registers a slight decrease in the production records of 2011, losses due to drought and low levels of precipitation during the growth period. The greatest loss is found in Diana monoecious variety (26.54-20.82%) followed by Zenit varieties (27.37-22.97%), Armanca (29.27-25.32%), Silvana (28.89-25.04%) and Denise (26.96-25.30%). Siccative hemp oil has a yellowish green color and an iodine index of 140–156 g I_2_/100 g oil. Hemp seed are rich in mineral based Ca (144–955 mg/100 g seed), Mg (237–694 mg/100 g seed), K (463–2821 mg/100 g seed), Fe (1133-2400 mg.kg^-1^), Mn (63–110 mg.kg^-1^) and Zn (42-94 mg.kg^-1^). For the soil the following macroelements concentrations were determined: Ca (2100–2520 mg.kg^-1^), Mg (320–376 mg.kg^-1^) and K (232–257 mg.kg^-1^). Mn (156–197 mg.kg^-1^) and Zn (54–67 mg.kg^-1^) remain within normal limits for Romania. The soils in the experience area contain large amounts of Fe (19000–20430 mg.kg^-1^). The presence of K in large quantities determines the accumulation of large quantities of Fe in the soil.

**Conclusion:**

Hempseed belonging to the five Romanian varieties are rich source of nutrients (Ca, Mg, K) and unsaturated oil easily digestible by the body, but the presence of Cd concentrations above the upper limit puts a question mark over the use of seeds in various food products. Hemp extracts easily certain metals from the soil. Significant amounts of Fe (1133–2400 mg.kg^-1^), Mn (63–110 mg.kg^-1^), Zn (42–94 mg.kg^-1^) and Cd (1.3-4.0 mg.kg^-1^) are found in hemp seeds. Hemp (*Cannabis sativa L*.) is included among plants suitable for phytoremediation of soil contaminated with cadmium, zinc and iron.

## Background

In recent years, the desire to adopt a healthy diet, draws attention to hempseed and food products derived from hempseed (oil, flour, milk, bakery products, chocolate, beer, etc.). Hemp (*Cannabis sativa L*.) is a highly variable crop with varieties both for fiber and oil. Hemp seeds and hemp oil have a high nutritional value and both are used for human consumption and animal feeding [1-3]. The nutritional value of hemp seeds is based on the content of protein and oil. Hemp oil is particularly valuable for the health because of its content in fatty acid, mostly being unsaturated acids, completed by high contents of phytosterols, but the properties of hemp oil are still little known in Romania [4-6]. The high price and reduced duration of keeping of the hemp oil translate in a low interest by the people for this alimentary supplement. Reduced supplies and the desire to get rich fast can bring a forged hemp oil on the market with an oil like the rapeseed oil. The authentication of the fatty acid in vegetable oils requires chromatography technique or FT-IR spectroscopy [7,8]. The 3:1 perfect ratio of omega 6 and omega 3 brings results as treatment for various diseases such as cardiovascular problems, reduction of fibrinogen (in atherosclerosis), in the production of prostaglandins series 3 (PG3) [9,10]. The reduction of the cholesterol is a major benefit of β-sitosterols present in the unsaponifiable of the vegetable oils like rapeseed, soybean and the hemp oil [11]. This acid γ-linolenic acid (GLA; 18:3ω6) in unsaturated oils such as hemp, flax or evening primrose oil refreshes and moisturizes the skin even with anti aging effects [12]. Fat calories in your diet should not exceed 2500 calories/day. The daily 9–18 g/day LA (18:2 ω6) and 6–7 g/day LNA (18:3 ω3) provides the optimum fatty acid ration the body requires [9,13].

Hemp is an excellent soil phytoremediation agent because it extracts heavy metals [14,15]. The use of hemp seeds in various derived foods and the consumption of supplements of hemp seeds bring attention to the potential negative effects of heavy metals possibly contained. Heavy metals are present naturally in soil in low concentrations. The presence of heavy metals in soil, as the natural background from pedogenetic processes or from human activities brings the current need to analyze their content in soil, water, air, crops and food. The capacity to absorb heavy metals depends on the geochemical characteristics of the soil and the nature of crops, some of them have a high potential to accumulate higher concentrations of heavy metals [15]. However, most crop plants can not survive on soils heavily contaminated with heavy metals [16].

Plants have different storage centers in vegetative and reproductive organs. Leaves can contain large quantities of metal, while the seeds have low metal amounts [17]. Thus, cereal grains accumulate low amounts of metals, while vegetables accumulate higher amounts especially in leaves [18,19]. Heavy metals are related to certain components of cell membranes, enzyme systems in the body or have the role of catalyst. Nutritional deficiency caused by lack of metals in food or the amount of metals exceeding a certain threshold, produces toxic effects. The implementation of procedures and control mechanism imposed by national and international regulations reduce considerably the maximum levels of toxic metals in food.

Hemp seeds are major sources of magnesium necessary for the body. Also, they bring a substantial contribution of phosphorus, iron and manganese [20]. Hemp (*Cannabis sativa L*.) along with rape or spinach is among the plants appropriate for decontamination of soil contaminated with Zn and Cd [21]. Hemp mainly extracts Ni concentrating it in leaves, not so much in the seeds [15]. Hemp is great consumer of nutrients (N, P, K), because during the growing season it develops a large vegetative mass and accumulates 70-75% of the dry matter in the first period of vegetation. The lignification process of the stem and the increase in resistance of the plant requires significant calcium intake. Potassium is also a key nutrient to ensure resistance to cold, drought and pests, but primary for the initial stage of seed formation. Magnesium is a component of oil and it contributes to the formation of hemp seeds [22-24]. Magnesium deficiency induces leaf chlorosis and the plants are small.

Manganese is an essential element in the activation of plant enzymes, it increases hemp plants resistance to drought and cold and helps to assimilate nitrogen from the soil. Increasing the concentration of manganese in the soil over 1500–3000 mg.kg^-1^ induce over 300–350 mg.kg^-1^ in the plant as a result of fertilization or due to pollution sources such as sewage sludges [24-26]. Excess manganese is found in acid soils (pH less than 5.5), with low organic matter and excess water. Iron is another plant nutrient involved in nitrogen metabolism and photosynthesis. The concentration of iron minerals in different layers of soil is ranged between 100–100000 μg∙g^-1^[27]. Fe and Mn intake by hemp seed must be within the upper tolerable daily intakes (UL) level of 45–11 mg/day/person [28]. Zinc is the cofactor of many enzymes, proteins and influences the electronic transfer in Krebss cycle reactions affecting the plant’s energy production. In accordance with the zinc transfer coefficient from soil to plants of 1–10 [29], the daily intake required by the plant is 70 mg/g Zn, and the critic one is 170 mg/g for soil containing 50–100 mg/g [30]. The recommended daily intake for adults is 8–11 mg Zn/day and an excess reduces the levels of high density lipoproteins and reduces the immune function [28]. Cadmium in soil poses a risk both for human receptors, as well as for the ecological ones, because of its relatively high toxicity and uptake in plants. Cd binds rapidly to the intra and extra cellular proteins, leading to the rupture of cell membranes and to the stoppage of the cells functions. As the most important natural cadmium sources we can mention integral cereal, potatoes and seafood. As a result maximum admitted cadmium content in cereal is 100 μg.kg^-1^ and in medicinal plants 300 μg.kg^-1^[19]. The medium Cd content in soil is of 0.5-0.8 mg.kg^-1^, and the maximum limit admitted for the total content of Cd tolerated by the plants is between 3–5 mg.kg^-1^, while in Romania the maximum concentration of Cd allowed in soil is 1 mg.kg^-1^[8,29,31,32].

## Results and discussion

### Oil content and fatty acids from whole hemp seeds

Table 1 presents the content of oil in analyzed monoic (Zenit, Diana, Denise) and dioic (Armanca, Silvana) hemp seeds obtained on parcels non fertilized N_0_P_0_K_0_ with a seeding spacing of 20 cm, in 2011 yield. The average content of oil in whole seeds of the Romanian hemp varieties analyzed is of 27.81% of the biological material used in planting and 23.89% for the production obtained in 2011. These values are slightly lower than the ones of 30-35% reported in literature [1,20,33]. The high monthly average temperatures (25–26°C) during the flourishing period and low rainfall level of 223 mm, result in an incomplete maturation of seeds and a decrease in oil content. Relative weight of 1000 seeds varies between 17 and 23 g and germination properties between 80-90%. The dioecious varieties have a higher oil content. The high content of unsaturated fatty acid in hemp oil is reflected in the value of the iodine index of over 140 g I_2_/100 g oil.

**Table 1:** Oil content^* ^**(%) in hemp seed varieties and iodine value**

**Samples**		**Zenit**	**Diana**	**Denise**	**Armanca**	**Silvana**
**Oil content % (w/w)**	**2010**	27.37 ± 0.11	26.54 ± 0.22	26.96 ± 0.06	29.27 ± 0.37	28.89 ± 0.08
	**2011**	22.97 ± 0.96	20.82 ± 0.85	25.30 ± 0.14	25.32 ± 1,21	25.04 ± 0.34
**Iodine value**		140.5 ± 1.50	141.3 ± 1.24	144.4 ± 1.41	155.5 ± 1.62	150.6 ± 1.36
**(g of I**_**2**_**/ 100 g of oil)**						
***Iodine value from the literature *(g of I**_**2**_**/ 100 g of oil)**		140-172^**^				
		154-165^***^				

^*^Values are means ± SD of five hemp (*C. sativa)* oils, analyzed individually in triplicate.

^**^Tabără, V, 2005 [41].

^***^Anwar et al. (2006) [42].

### Metal content in soil and hemp seeds

**Figure 1:**
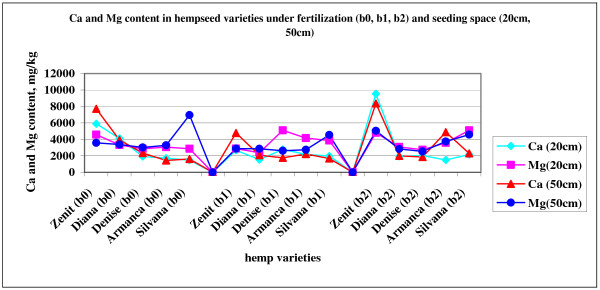
Ca and Mg content in hempseed varieties under fertilization (b0, b1, b2) and seeding space (20cm, 50cm).

The experimental site is located in the steppe zone with a high hidrostatic level of phreatic water represented by slightly salted chernozems, weakly acid on areas, and well supplied with phosphorus, humus, nitrogen and potassium. The levels of soil metals (Ca, Mg, K, Fe, Mn, Zn and Cd) from these areas are presented in Table 2. From total content of metals in soil, only a small part is available. Metal availability strongly depends on pH, which is influenced by the level of mineral fertilization. Calcium is present in adequate amounts in most soils. Calcium is a component of several primary and secondary minerals in the soil, which are essentially insoluble for agricultural considerations. These materials are the original sources of the soluble or available forms of Ca. Calcium is also present in relatively soluble forms, as a cation (positively charged Ca^++^) adsorbed to the soil colloidal complex. The ionic form is considered to be available to crops. Acid soils have less Ca, and high pH soils normally have more. As the soil pH increases above pH 7.2, due to additional soil Ca, the additional “free” Ca is not adsorbed onto the soil [34]. Much of the free Ca forms nearly insoluble compounds with other elements such as phosphorus (P), thus making P less available. Being a major cation, calcium availability is related to the soil cation exchange capacity (CEC), and it is in competition with other major cations such as sodium (Na^+^), potassium (K^+^), magnesium (Mg^++^), ammonium (NH4^+^), iron (Fe^++^), and aluminum (Al^+++^) for uptake by the crop. High K applications have been known to reduce the Ca uptake in plant. The soil on which fiber hemp was cultivated has medium available calcium content (Table 2). Fiber hemp does not have high requirements for calcium, the quantity found in soil is sufficient for hemp nutrition. The highest calcium content of hempseed was found in Zenit variety (9547 mg.kg^-1^), while the lower was found in Armanca variety (1440 mg.kg^-1^) (Figure 1). In Zenit and Silvana variety, fertilization with Fertileader Viti B:P:K=1:6:12 (factor- b_2_) leads to highest calcium content of hempseed. In Diana variety the highest calcium content was determined in unfertilized variant. In this case absorption of potassium applied as foliar fertilizer could block calcium absorption in hemp and lead to low calcium content of hemp seed.

**Table 2:** Content of Ca, Mg, K and heavy metals (Fe, Mn, Zn and Cd) of gleyed chernozem moderately low on hemp cultivation area dioecious and monoecious hemp

***Nr crt***	***Elemente *mgKg**^**-1**^	**Gleyed chernozem moderately weak dioecious hemp cultivation area**	**Gleyed chernozem moderately weak monoecious hemp cultivation area**	Normal metal contents, for Romania (NC)^* ^**mgKg**^**-1**^
*1*	*Ca*	*2100*	*2520*	*-*
*2*	*Mg*	*320*	*376*	*-*
*3*	*K*	*232*	*257*	*-*
*4*	*Fe*	*19100*	*20430*	*-*
*5*	*Mn*	*156*	*197*	*900*
*6*	*Zn*	*54*	*67*	*100*
*7*	*Cd*	*3,77*	*3,23*	*1*

^*^Order 756 from 16.08.2004 [32].

Using the F test (F_(4,24)_=26.5, p<0.05, F_5%_=2.78) it can be observed that the variety and the fertilization interaction with the variety (F_(8,24)_=4.1, p<0.05, F_5%_=2.34) determines significant differences in the Ca content from the studied hemp seeds.

By comparing the two average concentrations of Ca in the seeds corresponding to the B independent variable (treatment) statistically significant differences are observed between the average Ca content from hemp seeds under the effects of fertilization with N87:P96:K0 + 3 Kg/ha Corona (factor-b_1_) and N68:P64:K16+ 3 L/ha Fertileader Viti (factor-b_2_) (Table 3).

**Table 3:** **Metal content for five varieties hemp seed under influence fertilization and seeding distance (mg.kg**^**-1**^**)**

**Factor A-variety**	**Zenit**		**Diana**		**Denise**		**Armanca**		**Silvana**		
**Ca ±SD**											
Fertilization	20cm	50cm	20cm	50cm	20cm	50cm	20cm	50cm	20cm	50cm	Average –factor B
b_0_	5887±356	7707±321	4120±151	3907±299	1913±95	2327±239	1713±101	1440±68	1473±53	1620±63	3211
b_1_	2667±163	4780±284	1533±109	2067±152	2773±130	1753±71	2180±98	2213±142	1973±91	1687±41	2363*
b_2_	9547±341	8360±329	1933±180	2000±173	2040±140	1866±84	1507±91	4873±377	2147±117	2293±119	3657*
Average factor A	6491		2593		2112		2321		1866		
maximum allowable limits MAL- Ca unregulated											
**Mg ±SD**											
b_0_	4573±258	3567±271	3313±194	3367±372	2847±93	3020±110	3060±66	3273±110	2853±185	6940±641	3681
b_1_	2880±139	2880±168	2373±173	2860±238	5093±579	2607±73	4153±145	2727±271	3860±231	4540±406	3397
b_2_	4793±325	5033±267	3080±123	2833±139	2733±142	2533±104	3567±240	3740±186	5093±137	4580±92	3799
Average factor A	3954		2971**		3139**		3420*		4644**		
maximum allowable limits MAL – Mg unregulated											
**K ±SD**											
b_0_	21260±1127	28207±1589	6820±258	5953±144	5273±168	6427±329	7127±475	7967±423	7327±335	19787±2018	11615
b_1_	20007±1141	9960±482	4627±442	5733±267	9040±418	5213±320	13007±1078	7460±235	9880±491	13520±955	9845
b_2_	20860±1257	13087±1700	5260±286	5767±375	5207±353	5300±481	8980±314	14900±1181	12653±1171	10500±887	10251
Average factor A	18897		5693		6077		9907		12278		
maximum allowable limits MAL- K unregulated											
**Fe ±SD**											
b_0_	2000±148	2400±237	1700±84	1680±90	1740±49	1667±115	1593±35	1620±67	1630±42	1587±62	1762
b_1_	1620±36	1133±79	1767±105	1680±99	1740±102	1580±115	1580±135	1640±114	1560±59	1540±46	1584
b_2_	1600±95	2400±224	1720±67	1700±81	1660±67	1660±74	158086	1620±66	1647±120	1610±135	1720
Average factor A	1859		1708		1675		1606		1596		
maximum allowable limits MAL- Fe unregulated											
**Mn ±SD**											
b_0_	97±3.7	69±2.2	80±7.6	86.±9.3	99±5.5	90±4.04	86±2.7	81±3.3	86±3.8	96±7.6	87
b_1_	75±5.9	63±0.9	94±4.7	95±10.2	106±4.3	84±3.2	75±1.6	75±4.5	83±4.8	83±8.7	83
b_2_	64±2.6	64±3.0	95±5.5	98±7.9	110±8.6	85±6.9	81±3.2	76±4.6	84±2.3	84±2.4	85
Average factor A	72		91		96		81		86		
maximum allowable limits MAL-Mn 300–350 mg.kg^-1^											
**Zn ±SD**											
b_0_	62±3.7	50±2.4	94±6.2	53±2.3	58±4.9	56±2.5	71±3.3	70±6.7	67±3.5	78±5.5	66
b_1_	42±2.8	47±2.5	56±3.0	58±4.7	62±2.7	53±2.2	76±5.8	60±6.3	64±6.0	69±5.8	59
b_2_	49±4.0	46±4.3	60±4.6	59±3.2	65±3.5	60±5.0	66±4.3	65±4.2	66±5.2	68±4.8	62
Average factor A	51		64		59		68		69		
maximum allowable limits MAL- Zn 170 mg.kg^-1^											
**Cd ±SD**											
b_0_	1.3±0.27	2.0±0.11	4.0±0.13	3.3±0.31	3.3±0.47	2.7±0.20	3.3±0.21	3.3±0.13	3.3±0.12	3.3±0.20	3.0
b_1_	2.0±0.05	2.0±0.23	2.0±0.06	2.7±0.13	2.7±0.17	2.7±0.06	3.3±0.31	4.0±0.30	4.0±0.32	4.0±0.38	2.9
b_2_	2.0±0.15	2.0±0.18	3.3±0.21	2.7±0.10	4.0±0.33	3.3±0.26	3.3±0.06	3.3±0.08	2.7±0.08	3.3±0.27	3.0
Average factor A	1.9		3.0**		3.1**		3.4***		3.4***		
maximum allowable limits MAL –Cd 0.300 mg.kg^-1^											

Values are means ± SD of five hemp seed (*C. sativa)* varieties, analyzed individually in triplicate.

Magnesium is a component of several primary and secondary minerals in the soil, which are essentially insoluble, for agricultural considerations. These materials are the original sources of the soluble or available forms of Mg. Magnesium is also present in relatively soluble forms, and is found in ionic form (Mg^++^) retained to the soil colloidal complex. The ionic form is considered to be available to crops. Being a major cation, Mg availability is related to the soil CEC, and it is in competition with other major cations such as calcium (Ca^++^), potassium (K^+^), sodium (Na^+^), ammonium (NH4^+^), iron (Fe^++^), and aluminum (Al^+++^). It appears that potassium is a stronger competitor with Mg than it is sometimes considered to be. Cambic chernozem on which experiments were made has good magnesium supply, ranged between 320–376 mg.kg^-1^. Ideal soil Ca:Mg ratio range between 5:1 and 8:1. Like in calcium case, Zenit variety, Armanca variety at 50 cm and Silvana variety at 20 cm had a higher magnesium content after fertilization with Fertileader Viti B:P:K=1:6:12. Diana variety, Denise variety at 50 cm and Silvana variety at 50 cm have the highest magnesium content in unfertilized variant. Although soil has a good magnesium supply, its uptake could be disturbed by calcium and potassium presence in soil. Application of Corona K (N:P:K=8:11:39) fertilizer lead to highest magnesium content in hempseed Denise and Armanca variety at 20 cm (Figure 1).

The statistical processing by the ANOVA method of the Mg results generated in the trifactorial experience with three repetitions shows the effects of the variety on the Mg content in the seeds. The average Mg content in the Silvana seeds is significantly higher thant the concentration in the Diana, Denise and Armanca varieties. Changing the treatment and the seeding spacing doesn’t produce significant differences of the Mg concentration in hemp seeds.

**Figure 2:**
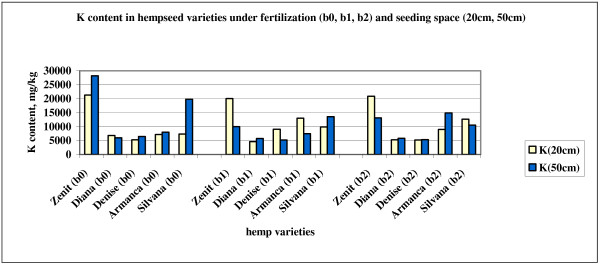
K content in hempseed varieties under fertilization (b0, b1, b2) and seeding space (20cm, 50cm).

Plant-available soil K is in the ionic (electrically charged) form. This charge is positive, making K a cation, represented as K^+^. Cations are attracted to, and held by negatively charged colloids (primarily clay and organic matter) that make up the cation exchange capacity (CEC) of the soil. The larger the CEC, the more K can be held by the soil and the more adequate it is for the plants. There are occasions when K uptake might be restricted due to an imbalance with other cation elements in the soil. For example, in many high pH soils there is an excess of Ca. Competition between Ca and K could reduce potassium uptake. Strongly acid soils will often have an excess of hydrogen (H), aluminum (Al), iron (Fe), and possibly other cation elements. These excess elements can compete with K for entry into the plant, and/or set up soil conditions that are unfavorable to efficient K utilization. It is generally accepted that there are some preferred general relationships and balances between soil nutrients. There is also a significant amount of work indicating that excesses and shortages of some nutrients will affect the uptake of other nutrients. Ideal ratio between Ca, Mg and K in soil is: Ca:K of 13:1, and Mg:K of 2:1. Soil used in experience has cation ratio thereabouts ideal. Potassium takes part in the formation of carbohydrates, enhances the sap’s circulation and creates hemp’s resistance to drought. Potassium excess enhances the autothinning phenomenon and increases the crop losses [35]. Potassium plays a vital role in: photosynthesis, translocation of photosynthates, protein synthesis, control of ionic balance, regulation of plant stomata and water use, activation of plant enzymes and, many other processes. Potassium is also known as the quality nutrient because of its important effects on quality factors such as size, shape, fiber quality and other quality measurements. The highest potassium content was determined in Zenit variety in unfertilized variant (21260 mg.kg^-1^ in 20 cm and 28207 mg.kg^-1^ in 50 cm) (Figure 2). Fertilization with both fertilizers gave different results concerning potassium accumulation in hempseeds. Potassium uptake by plants is affected by several factors. Higher soil moisture usually means greater availability of K. Increasing soil moisture increases movement of K to plant roots and enhances availability. Researches has generally shown more responses to K fertilization in dry years. Air is necessary for root respiration and K uptake. Root activity and subsequent K uptake decrease as soil moisture content increases to saturation. Root activity, plant functions, and physiological processes all increase as soil temperature increases. This increase in physiological activity leads to increased K uptake. Availability of soil K is reduced in no-till and ridge-till planting systems. The exact cause of this reduction is not known. Results of research point to restrictions in root growth combined with a restricted distribution of roots in the soil.

The experimental values F calculated for the separate action of the factors and their interaction in this experiment proves the fact that the variety (F_(4,24)_=17.8, p<0.05; F_1%_ =4.22) and the variety’s interaction with the seeding distance (F_(4,30)_=4.4, p<0.05; F_1%_ =4.02) have a significant influence on the K content from the analyzed seed samples.

After the use of the Tukey test the differences of the K concentration are considered significant for the Armanca –Denise(*), high significant for the Armanca-Diana (**) and extremely significant for the Silvana –Diana and Silvana –Denise (***) using as control the Zenit variety.

**Figure 3:**
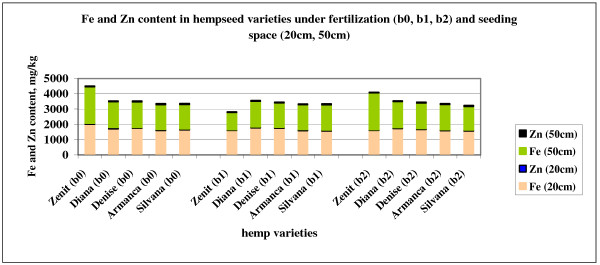
Fe and Zn content in hempseed varieties under fertilization (b0, b1, b2) and seeding space (20cm, 50cm).

Iron content of cambic chernozem is ranged between 19000 and 20430 mg∙kg^-1^, which means a good soil supply. Iron content of hempseed is relatively uniform in all varieties. Differences between fertilization variants are insignificant. Although the highest iron content was observed in Zenit variety (Figure 3). Iron uptake in hemp is inhibited by excessive amounts of soluble P, or high rates of P fertilizer. Increased NO_3_ -N uptake in plant due to fertilization can reduce Fe uptake by causing an anion-cation imbalance in the plant. Zn deficiency has been shown to increase the Fe uptake of many crops, sometimes to the point of toxicity. Conversely, high Zn availability reduces Fe uptake. It is well documented that iron and manganese are antagonistic, and one will inhibit the uptake of the other. K appears to play a very specific, but poorly understood role in the utilization of Fe by fiber hemp. Some research indicates that low K availability can result in increased Fe uptake in plant.

After the analysis of variance for the separate action and interaction of the factors it can be observed that neither the variety nor the seeding distance or the applied treatments have no influence on the Fe concentration in studied the hemp seeds.

Zinc content of soil is medium (Table 2), cambic chernozem having a good supply in organic matter which can chelate inorganic sources of Zn and increase their availability to fiber hemp. Zinc availability to hemp can be decreased by phosphorus fertilization. Plant roots appear to absorb Zn and Cu by the same mechanism. This causes interference in the uptake of one when the other is in excess in the root zone. Application of Fertileader Viti B:P:K=1:6:12 fertilizer lead to a decrease of zinc content in all hemp varieties, excepting Denise variety. In Zenit and Silvana variety and also in Diana variety at 20 cm, highest zinc content of hempseed was calculated in unfertilized variant. In this varieties application of fertilizers containing phosphorus inhibited zinc absorption by plant and its translocation to seeds. In only one variant, Armanca variety at 20 cm, application of Corona K (N:P:K=8:11:39) fertilizer containing zinc, lead to increase of zinc content.

By examining the factors and their interaction in the ANOVA factorial test of the Zn concentrations, it can be observed that the F values calculated are smaller that the theoretical ones F_5%_. So the differences between the averages are insignificant.

Comparing the Zn content in two varieties by the limit differences bring to attention the Zenit variety which has lower Zn content than Armanca and Silvana (*).

**Figure 4:**
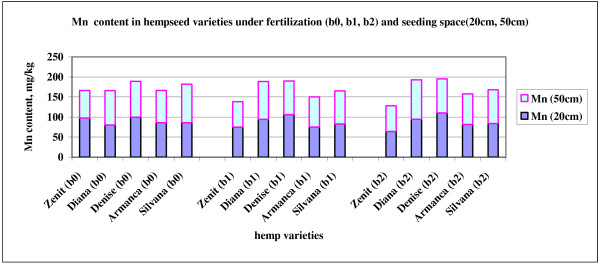
Mn content in hempseed varieties under fertilization (b0, b1, b2) and seeding space (20cm, 50cm).

Manganese content of experimental soil is low. Mn can be “tied up” by the organic matter such that high O.M. soils, like cambic chernozem, can be Mn deficient. Also soils high in available iron, or high iron applications can reduce Mn uptake in hemp plant. The lowest manganese content was determined in Zenit variety, after fertilization with Fertileader Viti B:P:K=1:6:12. Manganese content higher than 14 ppm is determined in Diana variety in both fertilization variants and in Silvana variety in 50 cm, unfertilized variant. Because of the soil low manganese content, most of the plant manganese comes from applied fertilizers (Figure 4). In varieties were highest content was determined in unfertilized plot, application of fertilizers containing phosphorus and potassium may inhibited manganese uptake by plants

As a conclusion for the statistical data determined through the ANOVA 5x3x2 technique, the different components of the independent variable (variety, fertilization and seeding distance) and their interaction doesn’t influence significantly the Mn content in the analyzed hemp seeds.

The F ratio of the independent variable and their interaction on the Mn content isn’t significant statistically, so as a result there aren’t differences between the averages of these variables.

Cadmium is a heavy metal known to have harmful effects on soil microorganisms and plants [36]. Experimental soil has higher content of cadmium, than admitted limit in soil (Table 2). Cadmium is often included in the fertilizer as a component of the phosphate component of the fertilizer. Removal of cadmium from the phosphate is considered expensive and rarely undertaken, so one of the cadmium source in soil could be fertilizers. The average content of Cd in the hemp seeds is ranged between 1.9 mg∙kg^-1^ (Zenit) and 3.4 mg∙kg^-1^ for the Armanca and Silvana varieties.

**Figure 5:**
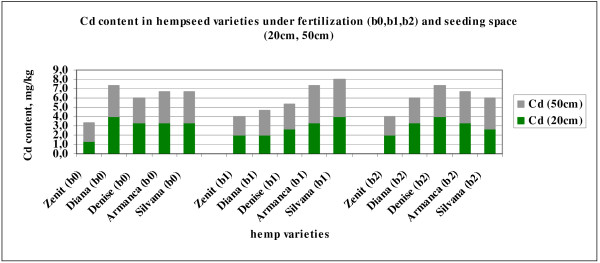
Cd content in hempseed varieties under fertilization (b0,b1,b2) and seeding space (20cm, 50cm).

Under the action of fertilization the Silvana and Zenit varieties show growths of the cadmium content of between 20 and 50% in the seeds of an N87:P96:K0 agrofield with Corona foliar fertilizer against an unfertilized one b_0_ (Figura 5).

After the analysis of variance (ANOVA one-way) with dependent variable of Cd showed that the variety, as an influence factor and the interaction of the variety with the seeding distance determines statistically highly significant differences (**) and extremely significant differences (***) of the Cd content in the seeds.

The researches made on typical chernozem soil moderately gleyed from Timisoara, at the same nitrogen doses, but in the presence of phosphorus and potassium fertilizers, at different levels, we observed a decrease of available content of Zn, Mn, Cu and Ni because of pH increase (that limit metal availability) and by the other hand, because of metal-phosphate combination which are forming in P excess condition. The most affected by mineral fertilization seems to be the available zinc content which decrease with almost 50% with increasing phosphorus and potassium doses. Available iron content increases with increasing the nitrogen, phosphorus and potassium. With increasing phosphorus and potassium doses, the available iron content decrease mainly because the formation of iron phosphates take place. The concentrations of monitored metals from hemp seeds studied under the influence of foliar fertilization and planting distance are found in Table 3.

## Conclusions

The Romanian hemp varieties can be the base of a diet containing lots of oils an a large intake of fatty acids. The utilization of hemp seeds and hemp seed by-products requires careful monitoring of metal content from soil and from the seeds. Hemp is one of the plants that extract easily metals such as Cadmium. P and K intake with foliar fertilizers like Fertileader Viti lightly enhances the average calcium concentration in seeds. Although over a certain value of K in soil the hemp will extract easier K cations instead of Ca. The increase of the K content in seeds determines a growth in the Mg content so that the K:Mg ratio in the monoic and dioic unfertilized and fertilized seeds are to be maintained in certain limits. Neither fertilizing nor distance don’t modify substantially the metal content such as Fe, Mn, Zn and Cd content.

### Experimental

#### Experimental site

**Figure 6:**
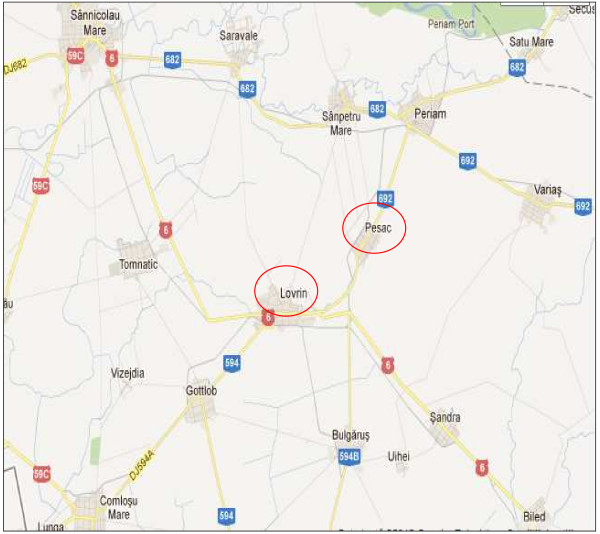
**Map of experimental sites (processed after Banat Map) **[40].

The experimental site is the Agricultural Research and Development Station in Lovrin, Timis area, located in the West of the Banat Plains, in the south west of Romania (45.548° N / 20.461° E (Figure 6). The agrochemical experiment took place in 2011 with five approved Romanian hemp varieties. The two experimental batches were placed in field under the form of a trifactorial experience, in divided plots, in three repetitions (Factor A- hempseed variety; Factor B - fertilization; Factor C - distance between plants d = 20 cm and d = 50 cm). Experimental lots as trifactorial experience in subdivided parcels, with dioecious hemp located on a typical chernozem soil moderately gleyed and the monoecious located in the north east of the village Lovrin to Pesac at a distance of 5 km, near irrigation canal Aranca. The average temperature during the growing period of hemp (April-September 2011) was around 19.5°C and the precipitation level has been 223 mm. The biological material used consists of three varieties of monoic hemp (Zenit, Denise and Diana) and two dioecious species (Armanca and Silvana). Two agrofields were achieved with complex fertilizers N16:P16:K16 and N18:P48:K0 using different doses, and 100 kg/ha NH_4_NO_3_. The effect of foliar fertilization with b_1_ - N87:P96:K0 + 3 kg/ha Corona K (N:P:K=8:11:39 and 0,1%B + 0,1%Cu + 0,1%Fe + 0,1%Mn + 0,1%Zn) and b_2_ - N68:P64:K16 + 3 L/ha Fertileader Viti B:P:K=1:6:12 were followed up in comparison to the control unfertilized b_0_ trials. Distance between rows was of 70 cm and the distance between plants was modified by 20–50 cm. Seeding rate was of about 3–5 kg/ha.

#### Samples collection and preparation

Soil samples were taken from the two sites with dioecious respectively monoecious hemp. Soil samples are air dried, ground and sieved through a 2 mm sieve. Hemp seed samples were taken from experimentally obtained seed production on the unfertilized parcel associated with each variety at a distance of 20 cm.

#### Hempseed oil content

Hemp seed oil extracted by Soxhlet method was with petroleum ether [37]. The fully automated Foss Soxtec 2055 system is used for fast and safe determinations of oil content of 6 samples of seed. Seed samples subjected to extraction are from yields of the five varieties of hemp produced in 2011, on unfertilized plots and sown at 20 cm, but also from the biological material used in seeding to achieve the experiment. Grind about 20 g of whole hemp seeds, mix and take three samples of 5.0 g powder which is subject to extraction with petrolium ether. Iodine value was determined in according by standard AOAC 920.158 methods [38].

#### Macro and microelements content

Soil samples, in three replicates for each variant, were air dried at room temperature, ground and sieved through a 2 mm sieve. From each soil sample 0.1 g was weight and subject it to mineralization with a mixture of HNO_3_ : HCl = 1 : 3 in the microwave with a method adapted after the 3050B method of the United States Environmental Protection Agency [39]. After mineralization the sample is diluted to 50 mL volume with 0.5N HNO_3_.

Samples of 3 g of ground seed were burned 8 h at 550°C in furnace (Nabertherm B150, Lilienthal, Germany). The ash was dissolved in HCl 20% and are brought to 20 ml in a volumetric flask.

The macroelements (K, Ca, Mg) and microelements (Fe, Cd, Zn and Mn) were determined by AAS (Varian 220 FAA equipment). The standard solution for calibration curve were prepared by diluting the standards (1000 mg/L). Mix standard solutions (ICP Multielement Standard solution IV CertiPUR) were purchased from Merck. Double distilled water was used for the preparation of reagents and standards. Concentrate nitric acid (HNO_3_ 65%), and concentrate HCl (37%), were obtained from Merck Germany. All chemicals were trace metal grade (Suprapur).

Method detection limits (MDL mg/L) for analyzed elements were: 0.02 mg/L for Mg and K; 0.06 mg/L for Fe; 0.03 mg/L for Ca; 0.04 mg/L for Zn; 0.01 mg/L for Cd and Mn. The average recoveries ± SD (%) for each element were: Fe (92±3.421%), Mn (95±0.897%), Zn (102±1.083%), Cd (105±0.764%), K (99±0.543%), Ca (92±2.121%), Mg (89±3.211%).

Metal concentration was obtained as the arithmetic average of three readings**.**

#### Statistical analysis

The results of the present study were processed by ANOVA one-way and the least significant difference test, in order to compare the mean values of the investigated parameters. The statistical processing and interpretation of the metal results of the trifactorial experience had as variation source hemp varieties, three different agrofonds, two seeding distances used and the three repetitions obtained after the parallel analysis of the samples. Computations Tukey post-hoc means comparisons and Levene’s test for equal variance was also included. Statistically significant differences are marked (*) and indicate a p value <0.05. Statistically highly significant differences are marked (**) and indicate a p value <0.01. Statistically extremely significant differences are marked (***) and indicate a p value <0.001. Statistical processing of data was performed using the Statistical Analysis System - SAS (Software Version 8.1. SAS Institute, Inc., Cary, NC).

## Competing interests

The authors declare that they have no competing interests.

## Authors’ contributions

MM, GP, EA, IR contributed equally to the study design, collection of data, development of the soil and vegetables sampling, analyses, interpretation of results and preparation of the paper. All authors read and approved the final manuscript.
